# The psychologically rich life questionnaire: Italian validation and exploration of its relationships with mindfulness, self-compassion, and cognitive fusion within the health psychology framework

**DOI:** 10.3389/fpsyg.2025.1525300

**Published:** 2025-02-20

**Authors:** Federica Mauro, Michela Di Trani, Luca Simione

**Affiliations:** ^1^Faculty of Medicine and Psychology, Sapienza University of Rome, Rome, Italy; ^2^Department of Dynamic, Clinical Psychology and Health, Faculty of Medicine and Psychology, Sapienza University of Rome, Rome, Italy; ^3^Department of Human and Social Sciences, Cultural Heritage, Institute of Cognitive Sciences and Technologies, National Research Council (CNR), Rome, Italy; ^4^Università degli Studi Internazionali di Roma, Rome, Italy

**Keywords:** mindfulness, health psychology, happiness, well being, self-compassion, cognitive fusion, psychological richness

## Abstract

**Introduction:**

Based on the theoretical framework that a fulfilling life encompasses happiness, meaning, and psychological richness, this study introduces the Italian translation of the Psychologically Rich Life Questionnaire (PRLQ-I), exploring its connection with mindfulness, self-compassion, cognitive fusion, and anxiety, positing psychological richness as a critical, yet distinct, component of well-being. Psychological richness, characterized by diverse and interesting experiences, complements the hedonic and eudaimonic dimensions of well-being, offering a broader perspective on what constitutes a meaningful life.

**Methods:**

After a subsequent back translation procedure, the resulting questionnaire version was checked for the comprehensibility of the items. Then, the PRLQ-I’s reliability and single-factor structure were evaluated through exploratory and confirmatory factor analyses in a large sample (*N* = 418) of the Italian population, as well as convergent and discriminant validity.

**Results:**

The exploratory factor analysis confirmed the original PRLQ structure, indicating one dimension, namely psychological richness, and the excellent internal consistency of the questionnaire. The confirmatory factor analysis revealed a good fit of the model for the one-factor structure. We then showed that psychological richness is positively associated with mindfulness and self-compassion, confirming that psychological richness is related to other happiness constructs but appears to be distinct from them. Moreover, psychological richness is inversely related to anxiety and cognitive fusion.

**Discussion:**

These results demonstrate the optimal reliability of the scale, even in its Italian version. They moreover provide suggestions for incorporating these related dimensions into programs designed to promote psychological richness, confirming its role in enhancing psychological well-being.

## Introduction

Health psychology is a field dedicated to understanding the interplay between psychological, behavioral, and social factors in health and illness (Taylor, 1990). Rooted in Engel’s biopsychosocial model (Engel, 1977, 1980), it emphasizes the importance of mental well-being in promoting overall health and preventing disease, recognizing that psychological states, such as happiness and life satisfaction, can significantly influence physical health outcomes (Diener and Chan, 2011).

Models of happiness and psychological well-being play a central role in health psychology. They provide a holistic framework for exploring how positive emotions and life satisfaction contribute to resilience, coping strategies, and healthier behaviors (Ryff and Keyes, 1995; Fredrickson, 2001). These models highlight how fostering positive psychological states can enhance individuals’ capacity to adapt to challenges, reduce stress, and promote long-term health and vitality.

According to the classical model proposed by Ryff (1995, 1998) psychological well-being is determined by two fundamental elements: happiness, or the hedonic dimension of life, and its meaning, or the eudaimonic dimension of life. More recently, the integration of a third dimension has been suggested: that of psychological richness, defined as the perception of a life characterized by a variety of interesting experiences and perspective changes (Oishi and Westgate, 2022). Previous works proposed it as a fundamental element of a “well-lived” life, highlighting its connection to happiness and the perception of an ultimate sense of life, but at the same time showing that it is related to different causes and consequences compared to these dimensions (Oishi and Westgate, 2022).

Diversity, fascination, and shifts in perspective that pave the way to wisdom have been suggested as the central attributes of psychological richness. This view is linked to the metaphor of the wanderer’s life as depicted in Nietzsche’s “Zarathustra,” characterized by amor fati (a love for fate), a willingness to experience life in all its facets, and an acceptance of the necessity of suffering and challenge as means to personal growth and the attainment of wisdom (Oishi and Westgate, 2022).

Also, in the health psychology biopsychosocial framework, the metaphor of the traveler’s life offers a valuable perspective (Bertini, 2012). Moving beyond the dichotomy of “normal” versus “deviant” in defining pathology, this image presents the individual as an active participant in their journey, encountering a mix of favorable and unfavorable events with a commitment to health and personal shaping of their journey. Here, health is defined as a “margin of tolerance toward the unfaithfulness of the environment” (p. 91), acknowledging life’s unpredictability and complexity, far from a linear progression. Illness represents a narrowing of this margin, indicating a reduced capacity to cope with environmental unpredictability, thus replacing the statistical concept of normality with normativity; in good health, life exhibits its inventive capacities and becomes a power to produce new norms.

Measuring psychological richness can thus offer an expanded perspective on well-being that extends beyond the confines of hedonic and eudaimonic measures, resonating with the principles of normativity, which underscore the significance of adaptability and the recalibration of personal norms, addressing both the complexity of human experience and the components central to personal growth and recovery in health psychology.

The Psychologically Rich Life Questionnaire (Oishi et al., 2019) is a standardized tool designed to measure the dimension of psychological richness in an individual’s life. The scale aims to capture the subjective perception of living a psychologically rich life, including the ability to adapt to new situations, learn from diverse experiences, and embrace the complexity of life, providing a reliable and valid measure that can be used in research and practice.

The present study aimed to validate the Italian version of the questionnaire, confirming its statistical properties, and to investigate possible factors that could be hypothesized to be positively correlated to psychological richness, such as mindfulness and mindful self-compassion, and negatively correlated, such as anxiety and cognitive fusion, in a wide sample of the Italian population.

### The role of mindfulness and self-compassion in psychological well-being

Happiness is commonly defined as a subjective experience characterized by positive emotions and life satisfaction. It includes both the presence of positive affect (e.g., joy, contentment) and the absence of negative affect (e.g., sadness, anxiety) (Diener, 1984). Diener et al. (1999) further describe happiness as a key component of subjective well-being, which reflects individuals’ overall evaluations of their lives, encompassing both emotional reactions and cognitive judgments about life satisfaction. Subjective well-being, as a broader concept, extends beyond momentary happiness and is described as “positive mental states,” including the full spectrum of evaluations—both positive and negative—that people make about their lives and their emotional responses to life experiences (Marks, 2024), thus capturing a more comprehensive and enduring sense of one’s subjective experience of life.

As previously mentioned, psychological well-being has been traditionally described as defined by two key dimensions: hedonic well-being, which centers on pleasure and happiness, and eudaimonic well-being, which focuses on purpose, meaning, and personal growth (Ryan and Deci, 2001). Expanding on this, Oishi and Westgate (2022) propose psychological richness as a third and distinct dimension of well-being that goes beyond the traditional notions of hedonic and eudaimonic well-being, emphasizing the role of complexity, variety, and depth of life experiences. According to Oishi and Westgate (2022), psychological richness reflects the dimension of an individual’s overall well-being related to exploration, curiosity, and a richer engagement with life, even in the face of challenges or difficulties. This perspective expands the traditional well-being framework, suggesting that a “rich” life filled with meaningful and diverse experiences can be just as valuable as a happy or purposeful life.

Since their definition, the relationship between mindfulness, self-compassion, and happiness has garnered significant interest in psychological research, highlighting a complex interplay that contributes to well-being and mental health. Mindfulness, defined as the awareness that arises through paying attention on purpose, in the present moment, nonjudgmentally (Kabat-Zinn, 1994), is posited to enhance an individual’s capacity to engage with experiences directly without overidentification, thereby reducing negative emotional responses and promoting happiness (see for example Crowley et al., 2022; Bajaj et al., 2022; Crego et al., 2021; Aldahadha, 2023).

Self-compassion, a concept closely related to mindfulness, involves treating oneself with kindness, understanding, and forgiveness, recognizing one’s experiences as part of the larger human experience, and holding painful thoughts and feelings in balanced awareness (Neff, 2003). Neff argues that self-compassion provides emotional resilience, allowing individuals to face difficult situations and emotions with a caring and accepting attitude, which, in turn, can enhance overall happiness. Empirical evidence consistently demonstrates a strong positive relationship between mindfulness, self-compassion, and happiness (Campos et al., 2015; Inam et al., 2021; Pastore et al., 2023). Interventions such as Mindfulness-Based Stress Reduction (MBSR) and Mindful Self-Compassion (MSC), alongside informal mindfulness practices, have been shown to significantly enhance overall well-being and happiness (Neff and Germer, 2013; Shankland et al., 2021; Rastelli et al., 2021). These approaches emphasize the therapeutic benefits of adopting a mindful attitude toward one’s experiences and fostering a compassionate stance toward oneself, leading to improved emotional well-being and life satisfaction.

Additionally, research highlights the crucial role of mindfulness and self-compassion in promoting positive psychological health, including greater happiness (Hollis-Walker and Colosimo, 2011). Recent longitudinal findings confirm a positive bi-directional relationship between self-compassion and happiness over time, with mindfulness identified as the most influential subcomponent of self-compassion in enhancing happiness (Pastore et al., 2023). These results suggest that mindfulness and self-compassion serve as mediators in the relationship between positive mental health outcomes and adaptive coping strategies, reinforcing their significance in fostering long-term psychological well-being.

On the other hand, the relationship between anxiety, cognitive fusion, and happiness is also a topic of growing interest within psychology, highlighting the intricate ways in which cognitive processes influence emotional well-being. Cognitive fusion, a central construct in Acceptance and Commitment Therapy (ACT; Hayes et al., 1999) refers to the tendency to become entangled with one’s thoughts and feelings in such a way that they dominate one’s behavior and perspective, often leading to psychological rigidity (Hayes et al., 1999). This entanglement with internal experiences can exacerbate anxiety, as individuals may struggle to distance themselves from distressing thoughts and emotions, leading to decreased happiness.

Anxiety, characterized by excessive worry, fear, and a heightened state of arousal, has been shown to impact happiness and life satisfaction negatively. The persistent and pervasive nature of anxious thoughts can lead to a cycle of cognitive fusion, where individuals become increasingly identified with their anxious thoughts and feelings, reducing their ability to experience joy and contentment (Greco et al., 2008; Cookson et al., 2020).

Cognitive fusion has been consistently identified as a key factor influencing psychological distress, anxiety, and overall well-being. Monestès et al. (2016) found that cognitive fusion mediates the relationship between psychological inflexibility and emotional distress, showing that individuals with high levels of cognitive fusion experience increased anxiety and reduced well-being. Similarly, Gillanders et al. (2014) reported a positive correlation between cognitive fusion and anxiety, alongside a negative correlation with quality of life. Their findings suggest that difficulty in distancing oneself from intrusive thoughts exacerbates anxiety and diminishes well-being. Supporting this evidence, McCracken and Vowles (2014) demonstrated that cognitive fusion is strongly associated with heightened psychological distress, including anxiety, and significantly lower levels of life satisfaction. Together, these studies highlight the detrimental effects of cognitive fusion on mental health and emphasize its relevance in therapeutic interventions.

Furthermore, interventions that target cognitive fusion, such as ACT, have been shown to reduce anxiety and increase happiness by promoting psychological flexibility. Psychological flexibility allows individuals to hold their thoughts and feelings lightly, reducing the impact of cognitive fusion on their lives (Hayes et al., 2006; Santos and Coutinho, 2024). By learning to observe their thoughts without becoming entangled in them, individuals can reduce the influence of anxiety on their well-being and enhance their capacity for happiness.

Overall, these findings clarify the relationships between the examined constructs and traditional notions of happiness. However, their specific connection to psychological richness remains underexplored, necessitating further investigation to deepen our understanding of the interplay between these dimensions and subjective well-being.

### The current study

Psychological richness adds another dimension to well-being, focusing on the complexity and variety of experiences rather than solely on positive affect or purposeful living (Oishi and Westgate, 2022). It highlights the value of engaging with diverse and complex experiences that foster personal growth, a nuanced understanding of oneself, and greater adaptability.

Mindfulness and self-compassion play key roles in this dimension by fostering emotional regulation, curiosity, and resilience (Tan et al., 2023; Van Dam et al., 2011; Ueno and Amemiya, 2024). Self-compassion and psychological richness share a reciprocal relationship, where novel experiences enhance coping skills and self-esteem, facilitating self-kindness (Liu et al., 2024). Additionally, previous findings underscored that fostering mindfulness and self-compassion in therapeutic interventions improves psychological health and promotes a richer engagement with life’s complexities, even in populations with significant mental health challenges (Mavituna et al., 2022).

By contrast, cognitive fusion is a key factor contributing to psychological inflexibility, as it prevents individuals from engaging in adaptive, value-driven behaviors by rigidly focusing on their internal experiences (Gillanders et al., 2014; Hayes et al., 2012). This dynamic is central to various forms of psychological distress, including anxiety and depression. Anxiety often leads to avoidance behaviors and a narrowed focus on perceived threats, which restricts the variety of experiences one might encounter (Barlow et al., 2002; Roberts et al., 2022).

In this context, we hypothesize that mindfulness and self-compassion positively influence psychological richness, whereas anxiety and cognitive fusion may hinder psychological richness by limiting one’s ability to engage with and appreciate diverse experiences.

## Methods

### Participants

The primary objective of this study was to validate the factor model of the Psychologically Rich Life Questionnaire through exploratory factor analysis (EFA) and confirmatory factor analysis (CFA). Determining an adequate sample size is challenging due to varying guidelines in the literature (Kline, 2016). Influencing factors include the complexity of the factor structure, the number of variables, desired statistical power, and significance level. Targeting a simple one-factor structure, we followed the recommendation of having over 100–150 participants (Cattell, 1978; Gorsuch, 1983). We aimed for a sample size of about 200 participants for both EFA and CFA, which was deemed sufficient for reliable results in a simple factor model (Guadagnoli and Velicer, 1988). This sample size is essential for complex factor structures or a high number of variables (Wang et al., 2013). Adhering to the suggested ratio of at least 10 cases per variable (Kline, 2016; Nunnally and Bernstein, 1967), our model with 17 observed variables requires a minimum of 170 participants. Our intended sample size of 200 participants comfortably meets this requirement, and the actual research included 418 volunteers (age 32 ± 10.34; 311 F), reinforcing the adequacy of our approach.

The study’s eligibility criteria required that individuals be 18 years or older and speak Italian as their mother tongue. About the education level, 2.8% of the sample reported to have primary school education level, 48.6% a high school level, 39.3% university degree level and the 9.2% doctorate or post graduated education level. 45.3% reported to have a dependent job position, 33.2% to be student, 9.2% to have an independent job position, 7.3% to be unoccupied, 2.4% to be retired, 1.4% to consider themselves as householders and 2.4% have other not mentioned occupation. Among students, 26.8% of the sample reported to be outside the prescribed time of the course. Finally, income situation of the participants was approximately evenly distributed among the revenue classes considered (0–5,000€/y: 19%; 5,000–12,000 €/y: 19.4%; 12,000–20,000 €/y: 24.9%; 20,000–30,000 €/y: 16.6%; over 30,000: 20.1%).

The total sample was then randomly divided into two subsamples using an algorithm in R (R Core Team, 2023), respectively, for EFA (*F* = 151; age 31.85 ± 10.46) and CFA (*F* = 156; age 31.71 ± 9.94). Convergent and divergent validity were assessed using the entire sample.

### Procedure

As a first step for the translation of the questionnaire in the target language, a subsequent back translation procedure was applied: the original questionnaire was translated from the source language into the target language by a bilingual translator, a native speaker of the target language with a deep understanding of both cultures; a second bilingual translator native speaker of the source language, not involved in the initial translation and unaware of the original text, translated the document back into the source language; the original document and the back-translated version were then compared by the original authors with the purpose is to identify discrepancies, misunderstandings, or cultural nuances that may have been lost or altered during the translation processes. Finally, the resulting Italian version of the questionnaire was administered to a sample of 10 subjects casually extracted from the Italian population to assess the grade of comprehensibility of each translated item. After answering the questionnaire’s items, the respondents’ level of comprehensibility for each item was expressed on a Likert scale ranging from 0 (not comprehensible at all) to 4 (totally comprehensible). All 17 translated items were reported to fall within the 3.5-point cut-off, confirming that the resulting version was suitable for administering to a larger sample. All data were collected through an online form shared on social media, and informed consent was obtained from all individual adult participants included in the study. All procedures performed were in accordance with the ethical standards of the institutional research committee at Sapienza University of Rome and with the 1964 Helsinki Declaration and its later amendments or comparable ethical standards.

### Measures

The Psychologically Rich Life Questionnaire (Oishi et al., 2019) is a standardized tool designed to measure the dimension of psychological richness in an individual’s life. It consists of 17 items that prompt respondents to reflect on the diversity of their experiences, the degree to which they have faced novel, challenging, or complex situations, and the extent to which they feel these experiences enrich their lives.

To confirm the factorial structure of the questionnaire, as performed in previous studies (Oishi et al., 2019), the Italian version of the PRLQ (PRLQ-I) was administered to our sample, along with three questionnaires classically used to measure well-being dimensions.

The Meaning in Life Questionnaire (MLQ; Negri et al., 2020) is a psychological assessment tool developed by Steger et al. (2006) to measure an individual’s perceived presence of, and the search for, meaning in life. The questionnaire is divided into two scales: the Presence subscale, which assesses the extent to which respondents feel their lives have meaning, and the Search subscale, which measures the drive and orientation toward finding meaning in one’s life. This self-report inventory consists of 10 items and utilizes a 7-point Likert scale ranging from “Absolutely True” to “Absolutely Untrue” for responses. It has been widely used in research and clinical settings due to its brevity and psychometric robustness.

The Satisfaction with Life Scale (SWLS; Di Fabio and Gori, 2020) is a psychological assessment tool designed to measure an individual’s global cognitive judgments of their life satisfaction. Developed by Diener et al. (1985), the SWLS is predicated on the theoretical understanding that life satisfaction constitutes a crucial component of subjective well-being. The scale comprises five items, which respondents rate based on a 7-point Likert scale ranging from strongly disagree to strongly agree, reflecting the extent to which they endorse each statement as applicable to their own life. The simplicity and brevity of the SWLS have facilitated its widespread use in research to gage overall life satisfaction as an indicator of subjective well-being, and it has been validated across a range of populations, demonstrating significant reliability and validity.

Finally, the Subjective Happiness Scale (SHS; Iani et al., 2014), developed by Lyubomirsky and Lepper (1999), is a psychological instrument designed to assess an individual’s perceived level of happiness. This self-report questionnaire consists of four items that measure global subjective happiness. Respondents rate their agreement with each item using a 7-point Likeness scale, where higher scores indicate greater happiness. The SHS has been validated across multiple populations and has shown consistent reliability and validity, making it a widely accepted measure in positive psychology research.

As previously mentioned, to evaluate possible correlations with the construct of psychological richness, we also used four different scales to measure the constructs of mindfulness, mindful self-compassion, anxiety, and cognitive fusion, respectively.

The Mindful Attention Awareness Scale (MAAS; Veneziani and Voci, 2015) is a psychological tool developed by Brown and Ryan (2003) that is designed to measure the trait of mindfulness. As defined in this context, mindfulness refers to the degree of attention and awareness an individual applies to present-moment experiences. The MAAS comprises 15 items that respondents rate on a 6-point Likert scale, ranging from “almost always” to “almost never.” Higher scores on the MAAS indicate greater levels of mindfulness. Numerous studies have validated the scale, demonstrating its reliability and validity across different populations and settings. It serves as a critical instrument for research in psychology, particularly in studies examining the role of mindfulness in well-being, stress reduction, and psychological functioning.

The Self-Compassion Scale (SCS; Petrocchi et al., 2014), developed by Neff (2003), is a psychological assessment tool designed to measure the construct of self-compassion. Self-compassion refers to being kind and understanding toward oneself in instances of pain or failure rather than being harshly self-critical. It also encompasses recognizing one’s experiences as part of the larger human experience and holding one’s painful thoughts and feelings in mindful awareness. The SCS is a 26-item scale where respondents rate items on a 5-point Likert scale from “almost never” to “almost always.” The scale includes subdimensions such as self-kindness, self-judgment, common humanity, isolation, mindfulness, and over-identification. Higher scores indicate greater self-compassion. The scale has been subject to extensive validation and is a reliable and valid measure across diverse populations and settings, contributing significantly to psychology and mental health research.

The State–Trait Anxiety Inventory (STAI; Pedrabissi and Santinello, 1989) is a psychological assessment tool developed by Spielberger et al. (1983), specifically designed to differentiate between the temporary condition of “state anxiety” and the more general and long-lasting “trait anxiety.” The STAI consists of two separate self-report scales: the State Anxiety Scale (Y-1) and the Trait Anxiety Scale (Y-2). The Trait Anxiety Scale (Y-2) focuses on measuring relatively stable aspects of anxiety, assessing how individuals generally feel across varying situations. This scale comprises 20 items that respondents rate on a 4-point Likert scale, ranging from “almost never” to “almost always.” Higher scores on the Y-2 scale indicate more significant levels of trait anxiety. The STAI, particularly the Y-2 scale, has been extensively validated and is widely used in clinical settings, psychological research, and health studies to measure anxiety levels, demonstrating high reliability and validity across different populations and cultural contexts.

Finally, the Cognitive Fusion Questionnaire (CFQ; Donati et al., 2021) is a psychological tool designed by Gillanders et al. (2014) to measure cognitive fusion, a key concept in Acceptance and Commitment Therapy (ACT). Cognitive fusion refers to the process of becoming entangled with one’s thoughts so that they dominate one’s behavior and experience, often leading to psychological inflexibility. The CFQ contains seven items that participants rate on a 7-point Likert scale, from “never true” to “always true.” Higher scores indicate greater levels of cognitive fusion, suggesting that thoughts are more likely to interfere with living by one’s values. The CFQ has been validated across various studies, showing strong reliability and validity. It serves as an essential measure for research and clinical practice within contexts that focus on understanding and altering the impact of cognitive processes on behavior.

### Data analysis

Due to objective constraints, all variables in this study were collected through participant self-reports, which could introduce common method bias in the relationships between variables. Following the recommendations of Zhou and Long (2004), procedural controls were implemented, such as ensuring participant anonymity and minimizing the participants’ ability to guess the study’s purpose. Harman’s single-factor was applied to detect common method bias in survey data to enhance the study’s rigor further. The test involves performing an exploratory factor analysis (EFA) on all the variables of interest without rotating the factors.

To validate the Italian translation of the PRLQ, as a first step for the Explorative Factorial Analysis (EFA), we performed the Kaiser-Meyer-Olkin (KMO) test to evaluate sampling adequacy by comparing the magnitudes of observed correlation coefficients to the magnitudes of partial correlation coefficients. We applied Bartlett’s Test of Sphericity to examine the hypothesis that considered variables are uncorrelated in the population. The EFA was conducted with no rotation, as we expect a single factor to emerge based on previous factor analyses on this scale. To decide the number of factors to extract, we inspected the scree plot and considered the Kaiser-Guttman rule (i.e., the number of factors to be considered equals the number of factors with eigenvalues greater than 1.0). Then, the factors individuated in the EFA were inspected at the item level, i.e., unrotated factor loadings and commonality, and their internal validity was assessed using Cronbach’s alpha and McDonald’s omega.

As a second step, we conducted a CFA using maximum likelihood estimation with robust (Huber-White) standard errors. The model was evaluated using four fit indices: relative chi-square (χ^2^/df), comparative fit index (CFI), root mean square error of approximation (RMSEA), and standardized root mean square residual (SRMR). We assumed as a good fit relative χ^2^ ≤ 3.00, CFI ≥ 0.90, TLI ≥ 0.90, RMSEA ≤ 0.05, and SRMR ≤ 0.08 (Hu and Bentler, 1999; Kline, 2016). The internal validity of the individuated factor(s) was then inspected. Also, the modification indices were inspected to determine the best-fitting model, allowing correlated error terms for some pairs of items. As a last analysis, we then examined the scale’s internal consistency by computing Pearson’s r to evaluate the strength and direction of the correlations between the PRLS score with other psychological variables, such as mindfulness, compassion, anxiety, cognitive fusion, and hedonic and eudemonic well-being, applying a Bonferroni *post hoc* correction to reduce the risk of type I errors when multiple pairwise tests are performed.

Finally, Average Variance Extracted (AVE)—a structural equation modeling (SEM) measure used to assess how much variance a construct captures from its indicators compared to the variance due to measurement error—and Composite Reliability (CR)—an SEM measure used to assess the internal consistency of indicators representing a latent construct—were computed to further assess convergent validity and reliability. Discriminant validity is supported when the variance captured by the construct (AVE)—in this case, psychological richness—exceeds the shared variance between constructs (squared factor correlation; Fornell and Larcker, 1981).

## Results

Harman’s single-factor test results indicated that 17 factors had eigenvalues greater than 1 when unrotated, explaining 67.77% of the variance. The first main factor accounted for only 34.85% of the variance, below the critical threshold of 40%. Therefore, this study does not suffer from significant common method bias.

The obtained KMO value of 0.914 indicates that the considered variables for the exploratory factor analysis share a substantial amount of common variance, making them suitable for the analysis, while the Bartlett’s Test of Sphericity result of χ^2^ ≤ 2475.326 (df.:136; *p* < 0.0001) suggests that our variables were significantly correlated and not orthogonal. This result, combined with a high KMO value previously mentioned (0.914), strongly supports the suitability of the data for factor analysis, indicating that the underlying assumptions for conducting an exploratory factor analysis are met.

**Figure 1:**
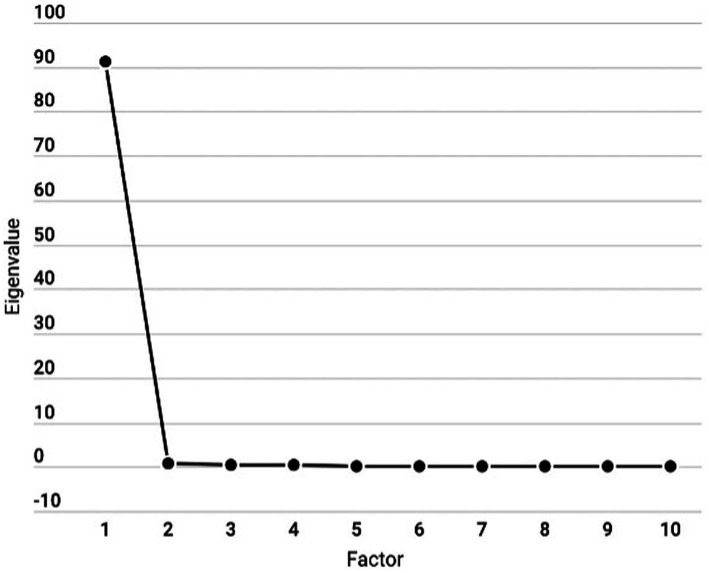
Scree plot showing the eigenvalues associated with each factor. The steep drop between the first and second factor suggests a single dominant factor, with minimal contributions from subsequent factors.

The application of Kaiser’s criterion revealed that in our data, just the first item directly measuring psychological richness appeared to get the indicated threshold, confirming the one-factor model hypothesized by the questionnaire’s original authors (see Figure 1).

Moreover, factor loadings (Table 1) were considered to evaluate the correlation between the variables and the factors extracted during factor analysis, showing a strong underlying dimension or construct within the collected data, given the substantial loadings for most variables and a high SS value (9.09) suggesting that the factor is explaining a significant amount of variance. Negative loadings were observed, as expected, only for the reverse items 14, 15, 16, and 17 (see Table 1).

**Table 1:** PRLQ original and translated items and their factor loadings.

Item	Translation	Factor loading
1	La mia vita è psicologicamente ricca *My life has been psychologically rich*	0.64
2	La mia vita è ricca di esperienze *My life has been experientially rich*	0.81
3	La mia vita è emotivamente ricca *My life has been emotionally rich*	0.67
4	Ho vissuto molte esperienze interessanti *I have had a lot of interesting experiences*	0.84
5	Ho vissuto molte esperienze nuove *I have had a lot of novel experiences*	0.80
6	La mia vita è stata piena di esperienze uniche e inusuali *My life has been full of unique, unusual experiences*	0.67
7	La mia vita consiste di momenti ricchi ed. intensi *My life consists of rich, intense moments*	0.82
8	La mia vita è stata emozionante *My life has been dramatic*	0.88
9	Faccio esperienze di una gamma completa di emozioni attraverso esperienze dirette, come viaggiare e andare ai concerti *I experience a full range of emotions via first-hand experiences such as travel and attending concerts*	0.67
10	Ho molte storie personali da raccontare agli altri *I have a lot of personal stories to tell others*	0.80
11	In punto di morte, molto probabilmente direi “Ho avuto una vita interessante” *On my deathbed, I am likely to say “I had an* *interesting life”*	0.84
12	In punto di morte, molto probabilmente direi “Ho visto ed. imparato molto” *On my deathbed, I am likely to say “I have seen and learned a lot*	0.78
13	La mia vita potrebbe essere un buon film o romanzo *My life would make a good novel or movie*	0.54
14	La mia vita è stata monotona My life has been monotonous	−0.73
15	Spesso sono annoiato dalla mia vita *I often feel bored with my life*	−0.58
16	La mia vita non è stata affatto movimentata *My life has been uneventful*	−0.69
17	Non riesco a ricordare l’ultima volta che ho fatto esperienza di qualcosa di nuovo *I cannot remember the last time I’ve done or experienced something new*	−0.62
SS		9.10

Finally, the internal consistency of the questionnaire, the degree to which a scale accurately establishes a cause-effect relationship among variables, minimizing the influence of confounding variables, was evaluated using Cronbach’s alpha and McDonald’s omega, showing excellent values (see Table 2).

**Table 2:** Cronbach’s alpha and McDonald’s omega for all administered questionnaires.

	Cronbach’s alpha	McDonald’s omega
**PRLQ-I**	**0.95**	**0.95**
MLQ	0.88	0.88
SWLS	0.90	0.90
SHS	0.90	0.90
STAI Y2	0.94	0.94
MAAS	0.88	0.88
SCS	0.94	0.94
CFQ	0.94	0.94

The results related to the PRLQ-I are highlighted in bold.

For the Confirmatory Factorial Analysis (CFA), a Maximum Likelihood Estimation (MLE) with a robust (Huber-White) standard error method was applied to the dataset. This sophisticated statistical approach is often used in structural equation modeling (SEM) and other complex modeling techniques. It is particularly valuable when the data must meet certain assumptions, such as normality. The robust standard errors adjust for potential violations, providing more reliable estimates and standard errors under such conditions.

The model fit evaluation using four indices—relative chi-square (χ^2^/df), Comparative Fit Index (CFI), Root Mean Square Error of Approximation (RMSEA) and Standardized Root Mean Square Residual (SRMR)—is a comprehensive approach that looks at different aspects of how well the model fits the observed data. The model demonstrated a generally good fit to the data, as evidenced by the CFI (0.941), SRMR (0.046), and a χ^2^/df ratio (203.45 / 104 = 1.96), indicating an acceptable to good fit. The RMSEA (0.071) suggests a fair fit, highlighting a potential area for improvement but not necessarily a critical flaw, given its closeness to the acceptable upper limit. The TLI value (0.875), while slightly below the preferred threshold, combined with the other indices, suggests that the model may benefit from some refinements. However, it is important to consider that, according to the complexity of the model, with a large number of parameters, achieving a perfect fit across all indices is challenging.

### Convergent and discriminant validity

Table 3 presents the correlation coefficients between Psychological Richness and the variables Meaning of Life, Satisfaction with Life, Subjective Happiness, Trait Anxiety, Mindfulness, Self-Compassion, and Cognitive Fusion. The correlational analysis yielded results in line with the expectations: the PRLQ-I’s assessment of psychological richness was found to be positively associated with the meaning of life (*r* = 0.33, *p* < 0.001), life satisfaction (*r* = 0.56, *p* < 0.001), and subjective happiness (*r* = 0.55, *p* < 0.001). This affirms previous observations within the Italian cohort, as noted in the research by Oishi and Westgate (2022). Additionally, the analysis upheld our original predictions, showing positive correlations between psychological richness and self-compassion (*r* = 0.40, *p* < 0.001), as well as mindfulness (*r* = 0.35; *p* < 0.001), while revealing negative correlations with anxiety (*r* = −0.47, *p* < 0.001) and cognitive fusion (*r* = −0.35, *p* < 0.001).

**Table 3:** Descriptive statistics and correlation analysis results of each variable.

	*M*	SD	PRLQ-I	MLQ	SWLS	SHS	STAI Y2	MAAS	SCS
PRLQ-I	78.32	21.46	**–**						
MLQ	33.86	5.46	0.33**	–					
SWLS	18.37	7.17	0.56**	0.24**	–				
SHS	3.91	1.41	0.55**	0.26**	0.62**	–			
STAI Y2	51.31	12.62	−0.47**	−0.17**	−0.73**	−0.58**	–		
MAAS	3.78	0.95	0.35**	0.13*	0.47**	0.33**	−0.67**	–	
SCS	3.62	0.80	0.40**	0.13**	0.59**	0.50**	−0.80**	0.60**	–
CFQ	32.95	11.1	−0.35**	−0.03	−0.58**	−0.41**	0.81**	−0.63**	−0.72**

*p < 0.05, **p < 0.01.The results related to the PRLQ-I are highlighted in bold.

Finally, the measurement model demonstrated good convergent validity and reliability, with an Average Variance Extracted (AVE) of 0.535 and a Composite Reliability (CR) score of 0.944. These values indicate that the AVE for the PRLQ-I scale exceeds the squared correlations of the constructs examined for discriminant validity.

## Discussion and conclusions

In 2019, Oishi and colleagues introduced the Psychologically Rich Life Questionnaire, a tool designed to measure the individual’s experience of a psychologically rich life, proposing psychological richness as another aspect of a good life, alongside happiness and meaning. The initial findings were replicated in diverse samples. The authors argued that while traditional measures of well-being focus on happiness and meaning, psychological richness captures a different aspect of a good life, including the pursuit of novel and diverse personal experiences, which some individuals value even more than happiness or meaning. This questionnaire can help understand and study this third aspect of a good life, providing a more comprehensive understanding of what makes life worth living.

The present study confirms the questionnaire’s excellent internal consistency consistent with earlier research. Both exploratory factor analysis (EFA) and confirmatory factor analysis (CFA) data affirm the one-factor model’s appropriateness for the Italian population, supported by a robust sample size. Moreover, the study highlights the questionnaire’s correlation with established measures of well-being, such as subjective happiness, life meaning, and life satisfaction, while simultaneously confirming the unique nature of psychological richness compared to these measures.

Confirming our hypotheses, the data further elucidate the PRLQ-I’s correlations with factors known to enhance subjective well-being, such as mindfulness and self-compassion, and its inverse relationship with elements that tend to undermine it, including anxiety and cognitive fusion, offering helpful directions for health promotion and psychotherapeutic interventions.

Psychological well-being has been increasingly recognized as a core objective in psychological care (Keyes, 2002; WHO, 2004). Contemporary perspectives define well-being not merely as the absence of illness but as an actively nurtured state of health (Ryff and Singer, 1998). This holistic view is now regarded as a crucial indicator of patient recovery and personal growth, valued equally alongside symptom reduction. Psychological well-being plays a preventative role by reducing the likelihood of new disorders and fostering resilience and long-term adaptive functioning (Keyes, 2007).

Within the health psychology framework, recent theories have emphasized adaptability as a core mechanism in maintaining a dynamic equilibrium of well-being (Cummins, 2000; Marks, 2024). These models draw from the biological principle of homeostasis, suggesting that individuals strive to stabilize their well-being despite external stressors through flexible and adaptive processes, thus underscoring the central role of adaptation and flexibility for well-being. The relationship between well-being and flexibility has been extensively shown in various contexts (see Woodruff et al., 2014; Howell and Demuynck, 2021; Guerrini Usubini et al., 2021; Russo et al., 2024), hinting at the importance of considering normativity over normality. Psychological richness may represent a distinct dimension of psychological well-being that is intricately linked to normativity, as it emphasizes the individual’s capacity to create, adapt, and redefine personal and social norms through engaging in diverse, complex, and meaningful experiences, thereby fostering growth and resilience.

In recent years, mindfulness-based interventions have been consistently spread due to their potential to build internal resources that support well-being and alleviate distress across multiple levels (Baer, 2003; Kabat-Zinn, 2003a,b; Zhang et al., 2021; Baer et al., 2012). These practices offer valuable pathways for health promotion and psychotherapeutic approaches, encouraging individuals to mindfully engage with life and embrace their full range of emotional experiences (Khoury et al., 2015; Bishop et al., 2004; Gu et al., 2015). Lately, this approach has been proposed as a foundation of happiness, conceptualizing happiness not as a fleeting emotional state but as a stable trait marked by a balanced and healthy spectrum of emotional responses over time (Van Gordon et al., 2023).

Aligned with this perspective, the data presented here suggest that mindfulness, self-compassion, and cognitive defusion may be key assets in cultivating a psychologically rich life journey. These resources may enable individuals to transcend a binary acceptance-rejection mindset toward life experiences, fostering a more normative state that embraces adaptability and resilience in diverse situations and appreciates them as fundamental aspects of a well-lived life. However, the specific pathways through which these psychological assets contribute to the cultivation of psychological richness remain to be further explored, highlighting the need for additional research to uncover their nuanced roles and interactions in promoting well-being.

### Limitations

This study acknowledges certain limitations within its sample. Firstly, the inclusion of age as a demographic factor is valuable for questionnaire validation, particularly when age-related dynamics are relevant to the constructs being measured. Our sample size is statistically adequate for the study’s objectives, with an average age of 32 ± 10.34 years, representing a middle-adult demographic. This is particularly meaningful for research focusing on mid-life transitions, health-related concerns in this age group, or generational comparisons. However, prior studies (Oishi et al., 2019) did not identify a significant correlation between age and psychological richness, suggesting that this construct may not be influenced by age. Secondly, the sample primarily comprised females. Nonetheless, earlier research (Oishi et al., 2019) that explored gender as a potential factor did not reveal significant differences, indicating that both men and women are equally likely to experience a psychologically rich life.

## Data Availability

The raw data supporting the conclusions of this article will be made available by the authors, without undue reservation.
